# Root system morphoanatomy of sunflower genotypes under water deficit

**DOI:** 10.1186/s12870-025-06468-z

**Published:** 2025-04-09

**Authors:** Orivaldo Benedito da Silva, Evaristo Mauro de Castro, Yohanna Vassura, Mateus Vilela Pires, Claudio Guilherme Portela de Carvalho, Luciana Marques de Carvalho, Marcio Paulo Pereira

**Affiliations:** 1 Laboratório de Anatomia Vegetal, Departamento de Biologia, Universidade Federal de Lavras, Lavras, Minas Gerais Brazil; 2 Empresa Brasileira de Pesquisa Agropecuária, Embrapa Soja, Londrina, Paraná Brazil; 3 Empresa Brasileira de Pesquisa Agropecuária, Embrapa Tabuleiros Costeiros, Aracaju, Sergipe Brazil

**Keywords:** Anatomical changes, Drought, *Helianthus annuus* L., Root development, Root system architecture

## Abstract

Sunflower is classified as a moderately drought tolerant crop. Genotypic variations and water availability are factors that influence the root development of the crop, which is important for water absorption in deep regions of the soil. Therefore, tests in controlled water deficit environments allow evaluating a set of morphoanatomical characteristics of the root system that attribute tolerance to water deficit, contributing to sunflower genetic improvement programs. The objective of this study was to identify a set of root morphoanatomical characteristics of four sunflower genotypes subjected to controlled water deficit. We tested four commercial sunflower genotypes (OLISUN03, AGUARÁ06, HELIO250 and BRS323) under well-irrigated (field capacity) and water restriction (40% of field capacity) conditions, completely randomized design with six replicates was applied, grown in rhizotron pot, allowing to evaluate root development through imaging and anatomical characteristics related to water absorption in different regions of the sunflower root system. Plants under water deficit showed changes that contributed to water absorption in different positions of root development. Under water deficit, the tissue differentiation occurred first near the root apex, while at field capacity differentiation occurred close to the root base. In the condition of water deficit, it was verified narrow root system architecture (RSA) for the genotype OLISUN03, deep RSA for BRS323, reduced endoderm thickness in OLISUN03 and vascular cylinder area in AGUARÁ06. In general, water deficit promoted changes in the morphological and anatomical characteristics of the root system. Morphological and anatomical modifications of the root system contribute to the anchoring and absorption of water and nutrients in places with little water availability in the soil.

## Introduction

The drought is part of a current climate change scenario that could be worsened in different parts of the world [1]. It is an environmental factor that causes damage to agricultural production. The morphoanatomical characteristics of the root system affectcontribute to the absorption of water by plants [2–3].

The root system comprises a set of characteristics associated with anchorage and uptake of water and nutrient from soil [4]. The water absorption and transport depends on the spatial distribution of the root system and anatomical characteristics, i.e. the root system architecture (RSA). It includes root positioning, length, angle, branching, surface area, coverage and diameter [5]. They are characteristics of tolerant or drought resistant plants [6]. Under water deficit, a root system with a narrow angle and deep growth is observed, enabling the absorption of water and nutrients in soil with low water availability. Observed in *Triticum durum* Desf. [7] and *Zea mays* L. [3].

Anatomical changes involved in the radial transport of water, such as apoplastic and symplastic pathways, occur in the root system [8]. The main changes are in the cortical parenchyma cells and apoplastic barriers, epidermis and sometimes exodermis and endodermis [9]. Other changes occur in the vascular cylinder, which is the tissue responsible for transporting water to the aerial part of the plant [9]. In this case, the main changes occur in the xylem vessels, which are modulated according to water availability, either in the diameter and number of vessels or in the thickness of the cell wall [9]. This set of modifications promotes better water absorption and efficiency, enhancing the productivity of crops such as *Glycine max* (L.) Merr. [10], *Arachis hypogaea* L. [11] and the reduction in xylem vessels, which prevents xylem embolisms, as evidenced in woody plants [12]. These are adaptive traits for capturing soil resources, such as water and nutrients, thus contributing to plant breeding programs [4–13].

Sunflower (*Helianthus annuus* L., Asteraceae) is an oilseed crop with a high oil content (40 to 60%) and protein content (17 to 20%) and with global potential for the production of edible oil and animal feed [14]. It is grown in regions with water restriction or supplemental irrigation [14] and under drought conditions, especially relative to other crops such as maize and wheat [15]. The root system plays a fundamental role in the development of the sunflower. It has long and deep roots, allowing the absorption of water and nutrients from the deepest layers of the soil [14–16]. However, water deficit in the vegetative, flowering and/ or achene filling phase promotes significant reductions in yield and oil percentage [15]. Therefore, identifying root characteristics that may contribute to drought tolerance and ensure yield is essential for crop breeding programs.

This study focus on contribute to sunflower breeding programs, by means study with four genotypes and a set of characteristics of the root system. Therefore, the hypotheses are as follows: (1) sunflower genotypes have a narrow root system able to uptake water in deep soil layers; (2) sunflowers under water deficit conditions promote root tissue differentiation closer to the root apex compared to those with adequate irrigation; and (3) under water deficit, sunflower genotypes, show increase in apoplastic barriers and a reduction in the vascular cylinder and xylem diameters. The objective of this study was to identify a set of root morphoanatomical characteristics of four sunflower genotypes subjected to controlled water deficit.

## Materials and methods

### Location and growth conditions

**Fig. 1:**
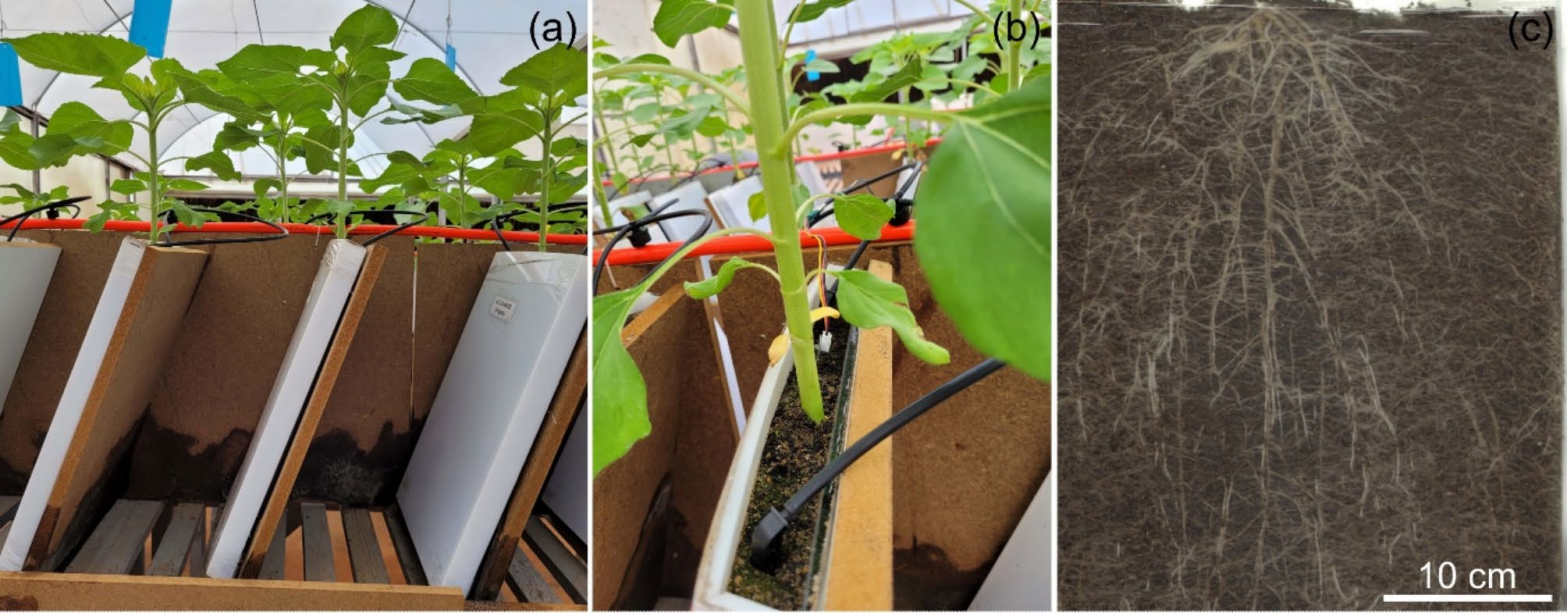
Sunflower plants in rhizotron pots (**a-b**) and visualization of the root system (**c**)

The study was conducted in a greenhouse located in the Botany Sector of the Department of Biology (DBI) of the Federal University of Lavras (UFLA), Lavras, MG, Brazil. During the experiment, plants were exposed to an average temperature of 26 ± 2 °C, monitored by a Sb-41 temperature sensor (Full Gauge Controls, Brazil), connected to the Tic-17RGTi thermostat (Full Gauge Controls, Brazil), controlling the greenhouse ventilation system. Relative humidity was between 50% and 70%, monitored with a “Max-Min Thermo Higro” digital thermometer and the average photosynthetic active radiation (PAR) was 652 µmol m^− 2^ s^− 1^, as measured in the plant canopy, using light sensor Li-190 (Licor., Inc., Lincoln NE, USA) and recorded on Li-1400 data logger (Licor., Inc., Lincoln NE, USA). The plants were exposed to a photoperiod of approximately 12 h of light and 12 h of darkness. The plants were kept in rhizotron pots (size: 42.5 × 29.5 × 3.5 cm) containing a transparent glass plate inclined at 43° towards the horizontal plane, promoting root development next to the plate and consequently facilitating the evaluation of the root system (Fig. 1). The pots were filled with 2.8 L of mixture of washed sand and the commercial substrate Tropstrato (vida verde^®^, Brazil) at a 1:1 ratio. The substrate had the following properties: pH CaCl_2_: 5.75; P: 65.70 mg dm^− 3^; K: 1.60 cmolc dm^− 3^; Ca: 23.80 cmolc dm^− 3^; Mg: 12.40 cmolc dm^− 3^; Al: 0.0 cmolc dm^− 3^; H + Al: 4.20 cmolc dm^− 3^; sum of bases: 39.80 cmolc dm^− 3^; cation exchange capacity: 42.10 cmolc dm^− 3^; base saturation (V%): 64.80; electrical conductivity: 1.5 mS cm^− 1^; density on a dry basis: 190 kg m^− 3^; density on a wet basis: 500 kg m^− 3^; moisture: 60% of the total weight of the substrate.

### Plants and experimental design

This study involved BRS323, a sunflower hybrid developed by Embrapa (Empresa Brasileira de Pesquisa Agropecuária, Brasília, DF, Brazil) and three commercial hybrids: OLISUN03 (Advanta Comércio de Sementes Ltda., Campinas, SP, Brazil), AGUARÁ06 (Atlântica Sementes, Curitiba, PR, Brazil), HELIO250 (Heliagro Agricultura e Pecuária Ltda., Araguari, MG, Brazil). The plants were obtained from seeds germinated on Germitest^®^ paper in a germination chamber at 25 °C under 12 h of light provided by lamps, reaching a PAR of 96 µmol m^− 2^ s^− 1^. After, they were transferred to the rhizotrons pots, when the rootlets reached approximately 2 cm in length.

The experiment was conducted with a 2 × 4 factorial design (two water conditions and four sunflower genotypes), and a completely randomized design with six replicates was applied, with one plant per rhizotron pot, totaling 48 plants. The water conditions evaluated included well-irrigated plants (WW), corresponding to 80% of field capacity, and plants under water deficit (WD), in which the field capacity was progressively decreased from 15 to 30 days after transplanting (DAT), up to 40% and maintained at that point until 51 DAT, i.e., the beginning of the reproductive stage. At this stage, it is possible to determine architectural and anatomical parameters of the root system involved in the effects of water deficit in the period that includes the beginning of flowering and the filling of the achenes. The field capacity was determined with the addition of water necessary to saturate the substrate (100% of the field capacity). Subsequently, the field capacity of the irrigated plants was calculated (80% of the field capacity), as well as the progressive decrease in the water deficit (40% of the field capacity). The moisture content of the compost in the rhizotron pots was monitored with soil resistive moisture sensors connected to the voltage comparator module (LM393) and regulated with a microcontroller (Arduino Mega2560). The sensor system was calibrated with information from each water condition, through Arduino Software programming (Arduino IDE 2.3.2). The irrigation system for each water condition was activated automatically, maintaining the moisture content of the substrate, determined for each water regime. In addition, the system consisted of an irrigation pump, distribution hoses and two drip pipes (length 15 cm) positioned at the upper edge of each rhizotrons pots. All plants were irrigated with nutrient solution at 40% ionic strength [17].

### Root angle

At 51 DAT, the vessels were scanned using an A3 scanner (1200 S, Mustek, China), and all analyses were performed using ImageJ^®^ software. The angle of the root system was obtained between the beginning of the main root at the upper edge of the compost and the limit of the secondary roots on the side of the rhizotron vessel. A representation of the root system architecture was created from six stacked images and the configuration of a time-lapse colour coder (LUT-Spectrum).

### Anatomical root analyses

**Fig. 2:**
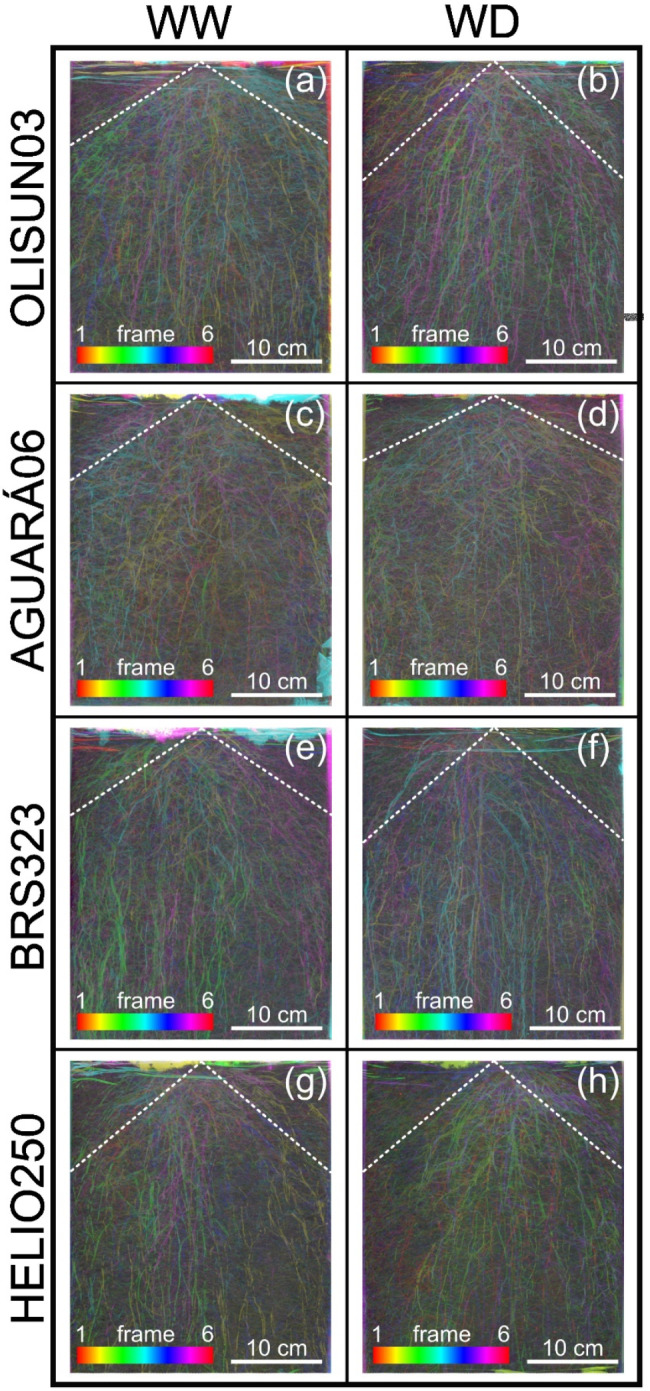
Representation of the root system angle of four sunflower genotypes grown in rhizotron pots under well-watered (WW) and water deficit (WD) plant conditions

At 51 DAT, the rhizotron vessels were disassembled, and roots approximately with 20 cm long were collected from the apex and fixed in 70% FAA solution (formaldehyde, glacial acetic acid and 70% ethanol, 1:1:18) by 72 h, after which they were transferred to 70% ethanol [18]. Subsequently, sections were obtained at 6, 12, 14 and 16 cm from the root tip towards the root base (Fig. 2) and dehydrated in increasing concentrations of ethanol (70, 80, 90 and 100%). At intervals of 2 h at room temperature, the cells were infiltrated for 24 h in historesin (Leica Microsystems, Heidelberg, Germany). The cross-Sect (7 μm thickness) were obtained using a semiautomatic rotating microtome, stained with 0.05% (w/v) toluidine blue [19] and mounted on permanent slides with Entellan (Merck, Darmstadt, Germany). The slides were photographed with a camera attached to a microscope (Eclipse E100-LED; Nikon, Tokyo, Japan). Quantitative anatomical data were obtained using ImageJ^®^ software, and analyses in which all tissues were differentiated were performed in 12 cm sections from the root apex (Fig. 2c). The thicknesses of the epidermis, exodermis, cortex, and endodermis were determined, as were the diameters of the metaxylem, vascular cylinder and cortex areas.

### Statistical analyses

The data were tested for normality using the Shapiro‒Wilk test. The means were subjected to analysis of variance (ANOVA) and when significant (*P* < 0.05), compared by the Tukey’s test for isolated factors (water conditions or genotypes) or interaction (water conditions × genotypes). The data presented demonstrate the interaction between the factors in the studies. In the absence of interaction between the factors, the data were presented separately for water conditions and genotypes, respectively. All analyses were performed using the software Sisvar 5.0 [20].

## Results

### Root angle

**Fig. 3:**
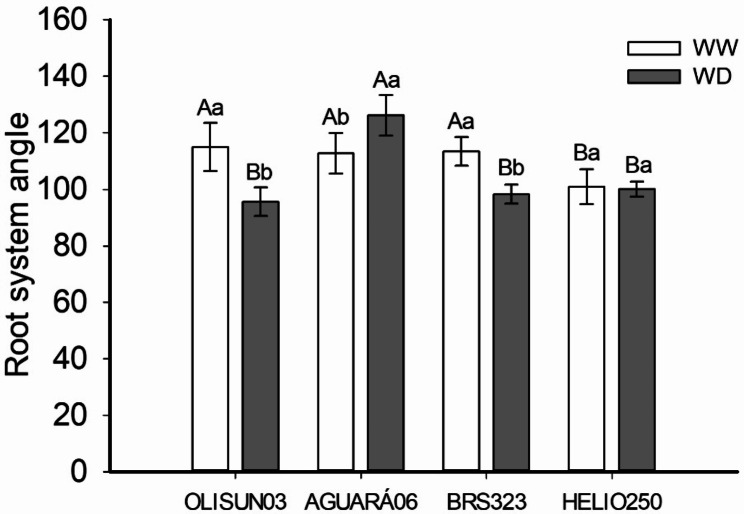
Root system angle averages of four sunflower genotypes grown in rhizotron pots under well-watered (WW) and water deficit (WD) plant conditions. Data are means ± SD. Means followed by equal letters, uppercase for genotypes and lowercase for water conditions do not differ by the Tukey’s test (*P* < 0.05)

Water restriction promoted a higher root system angle in the AGUARÁ06 genotype (Figs. 3 and 2c and d) and a reduced root system angle in the OLISUN03 and BRS323 genotypes (Figs. 3 and 2a, b, e and f). At field capacity, the HELIO250 genotype had a lower root system angle (Figs. 3 and 2g), while in the water deficit condition, the smallest root system angles occurred under OLISUN03, BRS323 and HELIO250 (Figs. 3 and 2b, f and h).

### Root anatomical characteristics

**Fig. 4:**
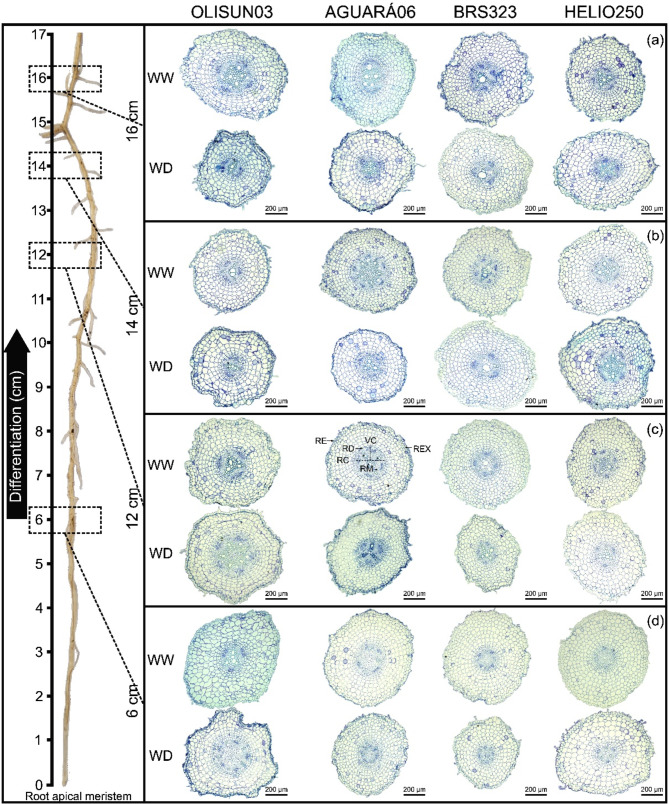
Transverse sections in different root regions four sunflower genotypes grown in rhizotron pots under well-watered (WW) and water deficit (WD) plant conditions. Root epidermis (RE), root exodermis (REX), root cortex (RC), vascular cylinder (VC), root endodermis (RD) and root metaxylem (RM)

There was a difference in the differentiation of root tissues between the two irrigation conditions, as observed in the anatomical sections collected in different positions, starting from the root tip (Fig. 4). In the plants under water deficit, all the root tissues located 6 cm from the apex were differentiated (Fig. 4d), while in those under field capacity, several tissues were differentiated 12 cm from the apex, especially those of the vascular cylinder (Fig. 4c).

**Fig. 5:**
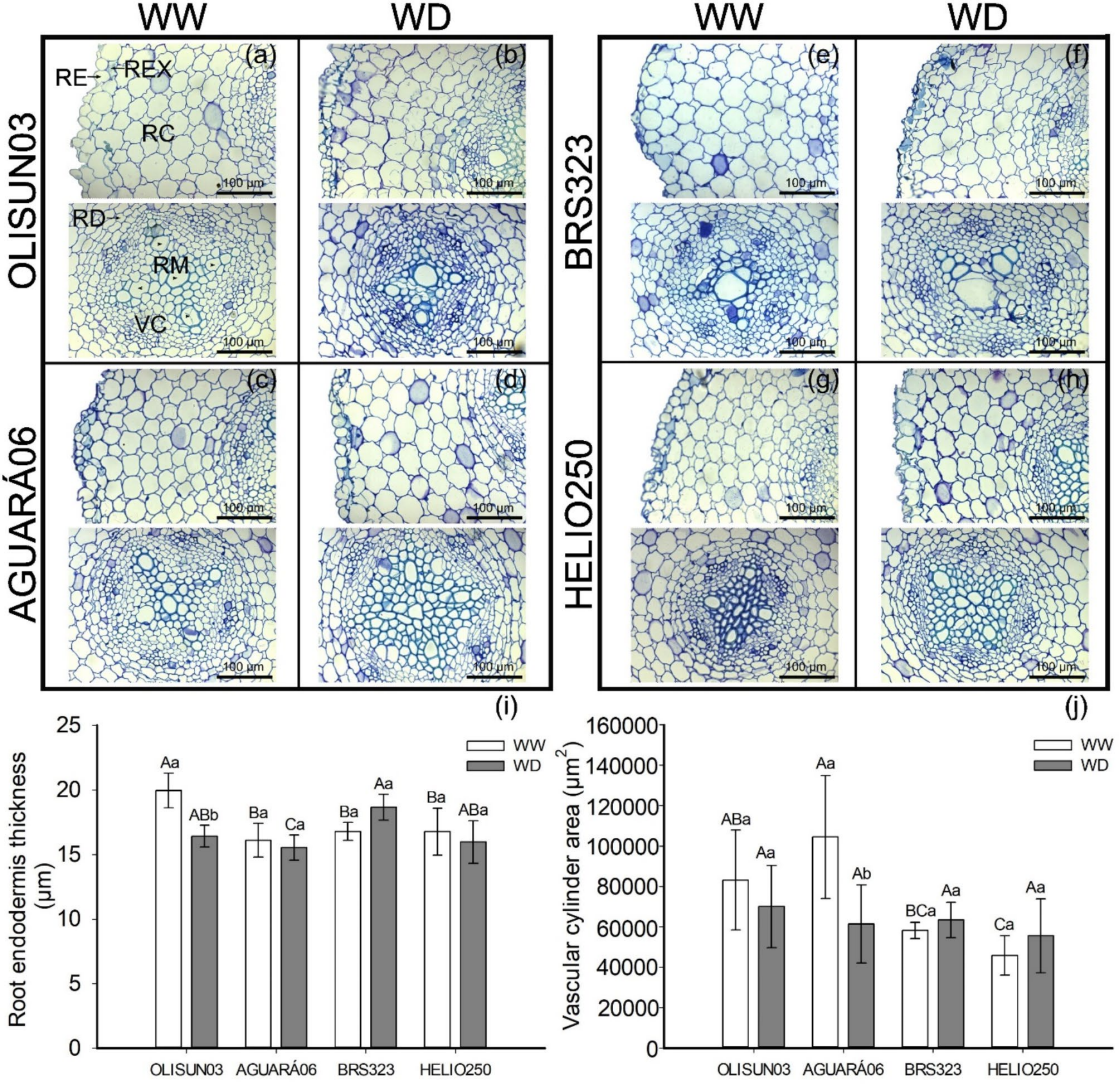
Transverse sections in roots of four sunflower genotypes grown in rhizotron pots under well-watered (WW) and water deficit (WD) plant conditions. Data are means ± SD. Means followed by equal letters, uppercase for genotypes and lowercase for water conditions do not differ by the Tukey’s test (*P* < 0.05). Root epidermis (RE), root exodermis (REX), root cortex (RC), vascular cylinder (VC), root endodermis (RD) and root metaxylem (RM)

The interaction between genotype and water condition had a significant effect on endoderm thickness and vascular cylinder area. The endoderm thickness was greater in OLISUN03 in the WW condition than in the other genotypes in the same condition; this was the only genotype for which a significant reduction was observed when the plants were subjected to the water deficit condition (Fig. 5a, i).

Comparing the genotypes under water deficit, it was observed that BRS323 presented greater endodermis thickness (Fig. 5e, i). Water deficit caused a reduction in the vascular cylinder area only in AGUARÁ06. Among the genotypes under the WW condition, the smallest areas of vascular casts occurred in BRS323 and HELIO250 (Fig. 5d, j).

There was no significant effect of interaction among the thicknesses of the epidermis, exodermis, cortex, and cortex area or metaxylem diameter. However, for all the genotypes, water deficit allowed for an increase in the thickness of the root epidermis and a reduction in the diameter of the metaxylem vessel, while the field capacity condition allowed for greater thickness and total cortex area (Table 1). Regardless of the water conditions, a reduction was observed in the thickness of the exodermis in OLISUN03 and in the diameter of the metaxylem in HELIO250.

**Table 1:** Root anatomical characteristics of four sunflower genotypes grown in rhizotron pots under well-watered (WW) and water deficit (WD) plant conditions

Water condition	RET (µm)	REXT (µm)	RCT (µm)	RCA (µm^2^)	RMD (µm)
WW	23.1 ± 2.2 b	21.8 ± 2.4 a	285.7 ± 23.7 a	473,726 ± 90,383 a	33.5 ± 5.1 a
WD	25.1 ± 2.9 a	20.0 ± 3.4 a	249.1 ± 22.1 b	382,047 ± 80,502 b	30.5 ± 4.4 b
Genotypes					
OLISUN03	22.8 ± 1.3 a	18.5 ± 2.4 b	267.0 ± 29.0 a	433,224 ± 67,955 a	34.4 ± 5.2 a
AGUARÁ06	24.9 ± 3.0 a	21.0 ± 3.8 ab	267.5 ± 30.1 a	474,036 ± 141,039 a	32.7 ± 3.5 a
BRS323	23.2 ± 1.7 a	21.1 ± 2.7 ab	274.3 ± 27.5 a	439,883 ± 65,669 a	35.6 ± 2.9 a
HELIO250	25.3 ± 3.8 a	22.9 ± 2.2 a	260.8 ± 19.5 a	364,403 ± 54,537 a	25.4 ± 3.0 b

Means followed by equal letters in water conditions and genotypes, respectively, do not differ by the Tukey’s test (*P* < 0.05). Means ± SD. Root epidermis thickness (RET), root exodermis thickness (REXT), Root cortex thickness (RCT), root cortex area (RCA) and root metaxylem diameter (RMD)

## Discussion

In general, plants under water deficit underwent root anatomical changes (Table 1). In this study, root anatomical characteristics involved in water absorption and conduction, such as endoderm thickness and vascular cylinder area, exhibited plasticity; that is, they responded to water deficit (Fig. 5). In particular, a reduction in the vascular cylinder was observed in the AGUARÁ06 (Fig. 5i) and in the endodermis in OLISUN03 (Fig. 5j). These adjustments in the endoderm allow a lower apoplastic barrier, contributing to the radial absorption of water. In addition, the reduction in the vascular cylinder is a protective mechanism and contributes to the movement of water into the shoots and the maintenance of ideal conditions for continued growth of the root system [2].

The angle occupied by the root system showed strategic water uptake by the root systems of sunflower genotypes subjected to controlled water deficit (Figs. 3 and 2). The efficiency of water uptake by the root systems of sunflowers belonging to different genotypes is related to the smaller angle of the root system, characterized as narrow and deep, as evidenced in the genotypes BRS323 and OLISUN03 under water deficit (Figs. 3 and 2a and f). These factors classify the sunflower as a moderately drought-tolerant crop, containing a deep root system, allowing it to absorb water in the deepest layers of the soil [14]. In addition, root development depends on factors such as genotypic variation and water availability in the soil [14–21]. The RSA is an important characteristic that determines the efficiency of soil water capture to prevent water stress in crops [4]. Crops under water deficit tend to develop roots with a narrower and deeper angle, thus allowing access to nutrients and water in deeper soil layers [7]. This response was observed for the genotypes OLISUN03 and BRS323 under water deficit.

Depending on the water conditions, the sunflower genotypes showed variation in the time of differentiation of the root tissues, observed in anatomical sections from the root apex to the root base (Fig. 4). Root development and differentiation of the vascular system are regulated by phytohormones, synthesized in the root cap and young shoots of the plant. Therefore, cytokinin (CK) and auxin (indole-3-acetic acid, IAA) regulate root development, vascular differentiation, and gravitropism and, together with ethylene, regulate lateral root initiation, responsible for determining root architecture [22]. In the plants under water deficit, there was total differentiation of tissues near the root apex (6 cm), while in those under field water capacity, there was differentiation even further away from the root apex (12 cm). Therefore, this differentiation is a strategy for capturing available water in the deepest part of the soil, evidenced by the narrow, deep root system and water absorption in this region of the rhizotron vessel, as observed in the genotypes BRS323 and OLISUN03 (Figs. 3 and 2b and f). Plants grown under abiotic stress promote anatomical structural changes in different regions of the roots; such an effect was observed for soybean under saline stress [*Glycine max* (L.) Merr.] [23] and for maize under high air temperature stress, drought stress or combinations of these two conditions (*Zea mays* L.) [24].

Plants grown under water deficit show morphoanatomical changes in the root system, evidenced in this study. Additionally, studies highlight the accumulation of reactive oxygen species (ROS), such as hydrogen peroxide (H_2_O_2_) and malondialdehyde (MDA); enzymatic activity, such as catalase (CAT) and ascorbate peroxidase (APX) [25], and an increase in the content of soluble sugar, sucrose, and starch [26], both in soybean roots under water deficit. These are rapid defense strategies against the effects of water deficit on the root system of cultivated plants, such as sunflowers.

The root system is important for support and supply of water and soluble inorganic mineral salts to the plant [26]. Anatomical characteristics of roots of cultivated plants, such as maize, have a strong relationship in the domestication process [27]. Therefore, information on the morphological and anatomical characteristics of the sunflower root system is important for the genetic improvement of the crop, thus contributing to productivity in the field. We highlight endodermis thickness in OLISUN03 (Fig. 5i), vascular cylinder area in AGUARÁ06 (Fig. 5j) and root system angle in OLISUN03 and BRS323 (Figs. 3 and 2). This information contributes to aspects of sunflower tolerance to water deficit, evidenced by long roots, absorbing water and nutrients in the deepest layers of the soil in drought conditions.

## Conclusion

In general, water deficit promoted changes in the morphological and anatomical characteristics of the root system. Under water deficit, OLISUN03 and BRS323 have smaller root system angles. Total differentiation of root tissues in water deficit occurred close to the root apex, and in irrigated plant conditions it occurred far from the root apex. Water deficit promoted a reduction in the thickness of the endodermis in OLISUN03 and a reduction in the area of ​​the vascular cylinder in AGUARÁ06. Morphological and anatomical modifications of the root system contribute to the anchoring and absorption of water and nutrients in places with little water availability in the soil.

## Data Availability

The datasets used and/or analysed during the current study are available from the corresponding author on reasonable request.
